# Complete Genome Sequence of *Klebsiella pneumoniae* Carbapenemase-Producing *K. pneumoniae* Myophage Miro

**DOI:** 10.1128/genomeA.01137-15

**Published:** 2015-10-01

**Authors:** Eleni M. Mijalis, Lauren E. Lessor, Jesse L. Cahill, Eric S. Rasche, Gabriel F. Kuty Everett

**Affiliations:** Center for Phage Technology, Texas A&M University, College Station, Texas, USA

## Abstract

*Klebsiella pneumoniae* is a Gram-negative pathogen frequently associated with antibiotic-resistant nosocomial infections. Bacteriophage therapy against *K. pneumoniae* may be possible to combat these infections. The following describes the complete genome sequence and key features of the pseudo-T-even *K. pneumoniae* carbapenemase (KPC)-producing *K. pneumoniae* myophage Miro.

## GENOME ANNOUNCEMENT

*Klebsiella pneumoniae* carbapenemase (KPC)-producing *K. pneumoniae* is a highly drug-resistant bacterium in the family *Enterobacteriaceae*. It can easily be spread in hospital settings, provoking deadly systemic infections (1, 2). This gives credibility to the prospect of bacteriophage-based therapy against the pathogen. Here, we describe the complete genome of pseudo-T-even myophage Miro.

Bacteriophage Miro was isolated from a sewage sample collected at College Station, TX, based on its ability to grown on KPC-producing *K. pneumoniae* strain A1. Phage DNA was sequenced in an Illumina MiSeq 250-bp paired-end run with a 550-bp insert library at the Genomic Sequencing and Analysis Facility at the University of Texas (Austin, TX). Quality controlled trimmed reads were assembled to a single contig of circular assembly at 47-fold coverage using SPAdes version 3.5.0 (3). The contig was confirmed to be complete by PCR using primers that face the upstream and downstream ends of the contig. Products from the PCR amplification of the junctions of concatemeric molecules were sequenced by Sanger sequencing (Eton Bioscience, San Diego, CA). Genes were predicted using GeneMarkS (4) and corrected using software tools available on the Center for Phage Technology (CPT) Galaxy instance (https://cpt.tamu.edu/galaxy-public/). Morphology was determined using transmission electron microscopy performed at the Texas A&M University Microscopy and Imaging Center.

Miro is a pseudo-T-even myophage with a 176.3-kb genome, a coding density of 95.6%, and a G+C content of 41.8%. This G+C content is much smaller than that of *K. pneumoniae*, which has an average G+C content of 57% (5). This suggests that Miro has adapted strategies to efficiently replicate its genome despite the lack of A and T nucleotides in the host (6). Miro contains one tRNA gene (Met) and six homing endonucleases, three of which have an AP2 domain (7). Of the 276 predicted genes in Miro, 183 were hypothetical conserved or novel. The other 92 were given a predicted function based on BLASTp and InterProScan analysis (8, 9). Miro is a member of the T4 subcluster I group described by Grose and Casjens (10). For annotation purposes, Miro has been opened to the *rIIb* gene, whose start codon overlaps with the stop codon of *rIIa*, such that the two genes cannot be separated, a common feature of pseudo-T-even phages (11). Miro is closely related to *Klebsiella* phage KP15 (accession no. NC_014036), with which it shares 94.5% nucleotide sequence identity across the genome. It also shares 92.9% nucleotide sequence identity across the genome with *Klebsiella* phage KP27 (accession no. NC_020080), as determined by Emboss Stretcher (12).

Like other pseudo-T-even phages, Miro contains several genes associated with base modification, most likely in order to hinder host restriction endonucleases (13). Also annotated were two RNA ligase genes that are used to repair tRNAs damaged by host defense systems. Miro encodes a T4-like NudE protein and has a bifunctional NMN acetylyltransferase/Nudix hydrolase gene found in all other subcluster I phages but not in T4 itself (14, 15).

### Nucleotide sequence accession number.

The genome sequence of phage Miro was contributed as accession no. KT001919 to GenBank.
